# MALDI-TOF MS combined with AUC method for tigecycline susceptibility testing in *Escherichia coli*

**DOI:** 10.1093/jacamr/dlad119

**Published:** 2024-03-07

**Authors:** Zelin Yan, Jiapin Li, Yanyan Hu, Yanyan Zhang, Yuchen Wu, Xiaoyang Ju, Chang Cai, Gongxiang Chen, Chengtao Sun, Rong Zhang

**Affiliations:** Department of Clinical Laboratory, Second Affiliated Hospital, Zhejiang University School of Medicine, Hangzhou, China; Department of Clinical Laboratory, Second Affiliated Hospital, Zhejiang University School of Medicine, Hangzhou, China; Department of Clinical Laboratory, Second Affiliated Hospital, Zhejiang University School of Medicine, Hangzhou, China; Department of Clinical Laboratory, Second Affiliated Hospital, Zhejiang University School of Medicine, Hangzhou, China; Department of Clinical Laboratory, Second Affiliated Hospital, Zhejiang University School of Medicine, Hangzhou, China; Department of Clinical Laboratory, Second Affiliated Hospital, Zhejiang University School of Medicine, Hangzhou, China; School of Veterinary and Life Sciences, Murdoch University, Perth, WA, Australia; Department of Clinical Laboratory, Second Affiliated Hospital, Zhejiang University School of Medicine, Hangzhou, China; Beijing Key Laboratory of Detection Technology for Animal Food Safety, College of Veterinary Medicine, China Agricultural University, Beijing, China; Department of Clinical Laboratory, Second Affiliated Hospital, Zhejiang University School of Medicine, Hangzhou, China

## Abstract

**Objectives:**

The wide spread of *tet*(X4) gene orthologues in the environment, food, poultry and humans is causing serious tigecycline resistance. Consequently, developing a fast and universal method to detect tigecycline resistance has become increasingly important.

**Methods:**

During 2019–2022, 116 *Escherichia coli* isolates were obtained from nine provinces in China. All isolates were tested for their susceptibility to antimicrobial agents by the microdilution broth method and for the *tet*(X4) gene by PCR. Ten *tet*(X4)-positive *E. coli* isolates were used to confirm certain conditions, including the optimal incubation time, the optimal concentration of tigecycline, and the cut-off of the relative growth (RG) value.

**Results:**

The optimal time and concentration of tigecycline for separation of susceptible and resistant isolates was 2 h and 4 mg/L, and the RG cut-off value was 0.4. We validated whether the experiment was feasible using 116 isolates of *E. coli*. The method yielded a susceptibility of 94.9% (95% CI: 81.4%–99.1%) and a specificity of 96.1% (95% CI: 88.3%–99.0%).

**Conclusions:**

This research has shown that this optical antimicrobial susceptibility testing method can rapidly differentiate between susceptible and resistant phenotypes in isolates of *E. coli.* In the same range as the current gold-standard methods, the clinical turnaround time is reduced from 48 h to 2.5 h. The above results suggest that the method has splendid specificity and operationality.

## Introduction

The *tet*(X4) gene orthologues are widely spread from a variety of sources, including the environment, food, poultry and humans. This widespread distribution of the *tet*(X4) gene has led to a worrying increase in resistance to tigecycline.^1,2^ Clinical bacteriology faces the challenge of selecting the right drugs for the appropriate patients at the right time, since the approval of tigecycline by the US FDA.^3–5^ The emergence and spread of tigecycline resistance, in both water sources and meat for consumption, have become an urgent problem for humans. Tigecycline is a derivative of minocycline, and it can overcome significant tetracycline resistance mechanisms.^6^ The *tet*(X4) family of genes encode flavin-dependent monooxygenases that enzymatically inactivate most tetracyclines, including tigecycline.^7^ Numerous tigecycline-resistant Enterobacteriaceae isolates have emerged not only from regions in China (CHINET, www.chinets.com) but also from Japan, Europe, North America and South America.^7–10^ This phenomenon indicates that resistance to tigecycline by *tet*(X4) variants has spread widely and been persistently propagated. The rise and spread of novel tigecycline resistance genes have seriously threatened the efficacy of tigecycline, such as to pose a looming threat to global public health.^11^

Around the world, several drug-resistant bacteria constitute a major issue, with *Escherichia coli* standing out.^12^*E. coli* are exposed to the effect of antimicrobial agents used in humans and animals, and the emergence and dissemination of resistance can occur, with antimicrobial-resistant bacteria being disseminated in the environment.^13^ Traditionally, *tet*(X4)-positive isolates can be detected using a number of genotypic and phenotypic methods, including agar dilution, disc diffusion, PCR and Etest methods. Disc diffusion and agar pore diffusion are relatively time-consuming methods (24–28 h). Moreover, methods such as disc diffusion and Etest are not reliable.^14^ There is now a published study showing that the modified disc diffusion method has a higher compliance rate, but it takes longer and has only been validated in *Klebsiella pneumoniae* and *Acinetobacter baumannii*.^15^ PCR genotyping is highly susceptible with very high specificity, but not always directly mirrored by the phenotype.^16^ Also, the operation involves rather complex technology that requires expensive reagents and instruments, and professional technicians.^17,18^ According to the CLSI and the EUCAST, broth microdilution (BMD) is the gold standard technique for testing tigecycline susceptibility. However, the above methods are time-consuming (exceeding 24 h–72 h), and have high operational requirements and high economic costs. Achieving acceptable susceptibility testing in clinical diagnosis and therapy is difficult due to the time-consuming nature of these procedures and the long turn-around time involved. In order to identify and test for *tet*(X4) gene-related tigecycline resistance, a test that enables both rapid and reliable identification is urgently needed. Therefore, the aim of this study was to find such a method for tigecycline susceptibility testing in *E. coli*.

Compared with other identification methods, MALDI-TOF MS has a turn-around time of 2.5 h for one isolate, including 2 h for the bacterial growth phase. The method has a small sample volume requirement and modest reagent costs. MALDI-TOF MS is being rapidly embraced by laboratories around the globe for developing antimicrobial susceptibility testing (AST) assays due to its rapid sample analysis speed and low operational cost. For the vast majority of data processing problems, simpler and more operable methods are needed in the era of big data. In this case, R software was used for digitizing the profiles of bacteria.^3^ Diagnostic test susceptibility and specificity are reflected in the receiver operating characteristic curve, and the AUC is an effective way to summarize the overall diagnostic accuracy of the test. Previous researchers have used AUC as a critical component in optimized, personalized vancomycin therapy for patients with MRSA invasive infections and assessing discrimination.^19,20^

In this study, the AUC was combined with the principles, characteristics and process of MALDI-TOF MS peptide quality fingerprinting based on protein levels to detect the resistance phenotype of *E. coli* carrying the *tet*(X4) gene. Based on this semi-quantitative drug susceptibility test method, it has been possible to detect isolates of the same complex from large-scale drug resistance testing.

## Materials and methods

### Bacterial isolates and culture

A panel of 116 *E. coli* isolates was included in the study. The isolates were collected from patients (24/116, 20.7%), animals (26/116, 22.4%) and the environment (66/116, 56.9%) from nine provinces of China from January 2019 to October 2022. Four of the 24 *E. coli* isolates (16.7%) examined in this study were found to possess the *tet*(X4) gene. The remaining 20 isolates (83.3%) were identified as *tet*(X4) negative. Ninety-two isolates of *E. coli* derived from animals and the environment were found to be positive for the *tet*(X4) gene. These isolates came not only from different geographical distributions but also were collected from different sources. The breakpoint concentration of 4 mg/L distinguished between resistant and susceptible isolates. Bacteria were incubated onto China Blue agar plates at 37°C overnight, and then were used for tests (Table 1).

**Table 1.** Detailed information about the 116 *E. coli* isolates

Source	Province	Gene	No. of samples
Human	Zhejiang	*tet*(X4)	4
	Zhejiang	—	1
	Shandong	—	4
	Henan	—	7
	Guangdong	—	2
	Fujian	—	3
Animal	Jiangsu	*tet*(X4)	10
	Shandong	*tet*(X4)	16
Environment	Zhejiang	*tet*(X4)	3
	Shandong	*tet*(X4)	7
	Henan	*tet*(X4)	16
	Guangdong	*tet*(X4)	10
	Hubei	*tet*(X4)	11
	Ningxia	*tet*(X4)	11
	Hunan	*tet*(X4)	8

“—” indicates no *tet*(X4) gene was detected for those isolates.

### AST

MICs of tigecycline for the isolates were determined by the broth microdilution method according to recommendations of the CLSI. However, due to the lack of established CLSI breakpoints for tigecycline, resistance was interpreted by breakpoints issued by the FDA (susceptible  ≤2 mg/L, intermediate 4 mg/L, resistant  ≥8 mg/L for MIC methods).^4^*E. coli* ATCC 25922 was used as a quality control strain in this study. For data analysis, the isolates with MIC  ≤2 mg/L were classified as susceptible and ≥4 mg/L as non-susceptible, based on the reference studies.

### Resistance profiling assay by MALDI-TOF MS

Proteins of *E. coli* isolates were extracted using an ethanol/formic acid extraction method.^21,22^ Fresh bacterial isolates were incubated in brain heart infusion (BHI) medium (HangZhou Binhe Microorganism Reagon Co. Ltd, China), and the cell suspension adjusted to maintain a cell density of 0.5 McFarland standard.^23^ Eight different cell suspensions were prepared, with 0, 1, 2, 4, 8, 16, 32 and 64 mg/L tigecycline (China National Institutes for Drug Control, China), respectively. These were incubated in a shaker at 200 rpm and 37°C for 1, 2, 3 and 4 h to determine the optimal time (2 h). After incubation, the suspensions were centrifuged (12 000×**g**, 2 min) to remove the supernatant, then washed once with 150 µL double distilled H_2_O, then 100 µL 70% alcohol was added. A further centrifugation (12 000×**g**, 2 min) was performed to remove the supernatant. Wash the tube and then remove the excess liquid inside the tube. Then add formic acid, acetonitrile and 0.4 mg/L internal standard RNase A enzyme 2.5 µL (40 mg/L; Sigma Aldrich, St Louis, MO, USA) and centrifug again (12 000×**g**, 2 min) to the mixture successively. A 1 µL sample of each *E. coli* protein extract was deposited on a MALDI-TOF target. Spots were overlaid with 1 µL of HCCA matrix solution (α-cyano-4-hydroxy-cinnamic acid matrix in 50% acetonitrile with 2.5% trifluoroacetic acid; Bruker Daltonik, Bremen, Germany). Data were collected for a mass range between 2000 and 20 000 Da, 240 laser shots, and at least one intensity measurement with a peak of 10^4^ or more (arbitrary units).^3^ An external calibration standard was used (bacterial detection standard; instruments were calibrated using Bruker Daltonik). Before identifying the specific peaks, the raw spectra were preprocessed by normalizing, smoothing and baseline subtraction.^24^ Uniformly compare it after exclusion of poor-quality spectra (poor-quality spectra can be defined as those lacking a peak at *m*/*z* 13 600 and a doubly charged ion at *m*/*z* 6800).^25^

### Statistical analysis and data evaluation

Automated analyses of the spectra were performed with a prototype software tool written with the freely available software package R.^26^ The software package R (Yang Xu, Shenzhen city Tencent computer system Co. Ltd, personal communication) calculates the AUC for each setting separately. The relative growth (RG) was calculated using the ratio of AUC of the same samples with and without tigecycline: RG = AUC_BHI + tigecycline_/AUC_BHI._^3^ We found the breakpoint concentrations of tigecycline and incubation times for each sample in preliminary studies: 4 mg/L for 2 h for tigecycline.

## Results

### Determination of the optimal incubation time and concentration

Initially, we selected two susceptible isolates and two resistant isolates to confirm the optimal incubation time. The tigecycline-susceptible isolates (ATCC 25922 and ATCC 8739) and resistant isolates (R753 and R655) were incubated with 32 mg/L tigecycline. We determined 2 h to be the optimal incubation time for this study.

**Figure 1.**
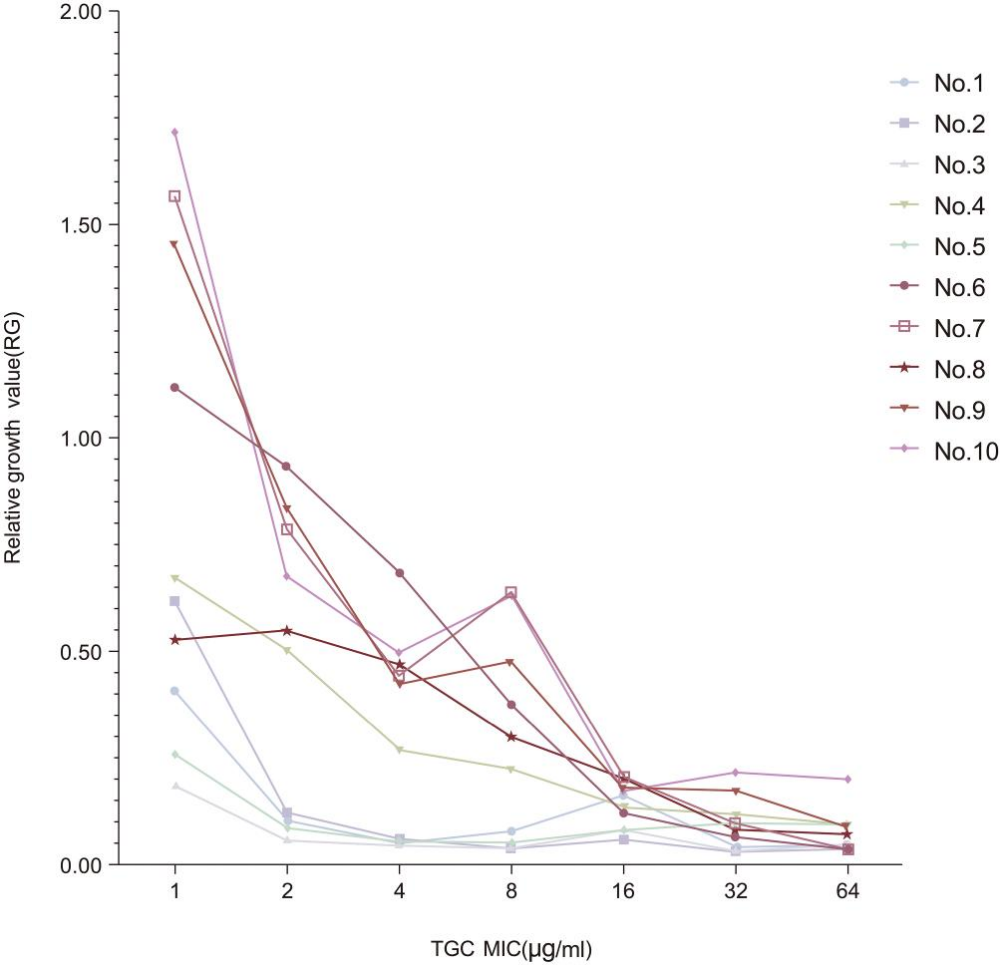
Plots of relative growth (RG) values of 10 *E. coli* isolates with MICs 0.25–16 mg/L incubated with tigecycline concentrations (1 mg/L, 2 mg/L, 4 mg/L, 8 mg/L, 16 mg/L, 32 mg/L, 64 mg/L). When the concentration of tigecycline was 4 mg/L, the separation of susceptible and resistant isolates was the most significant in all tested isolates of *E. coli*.

After 2 h of incubation, the relevant growth status of *E. coli* was quantified by the patterns in the presence of 32 mg/L tigecycline. Resistant and susceptible isolates were clearly distinguished (Figure 1).

For larger data analysis, automated software tools are more practical because a large number of graphs need to be analysed in the experimental verification. An automated R analysis of a large number of bacterial profiles was conducted using R software. The RG values of 10 isolates of *E. coli* with different MICs of TGC after 2 h incubation in the presence or absence of tigecycline (4 mg/L) are shown in Figure 1. The susceptible isolates showed RG values ranging from 0.1 to 0.4, and the resistant isolates had values approximately from 0.4 to 1.8, in all analyses. In summary, the sensitivity and resistance to the drug were clearly distinguished by a cut-off value of 0.4 in our study.

### MIC and PCR results were verified for the isolates

**Figure 2.**
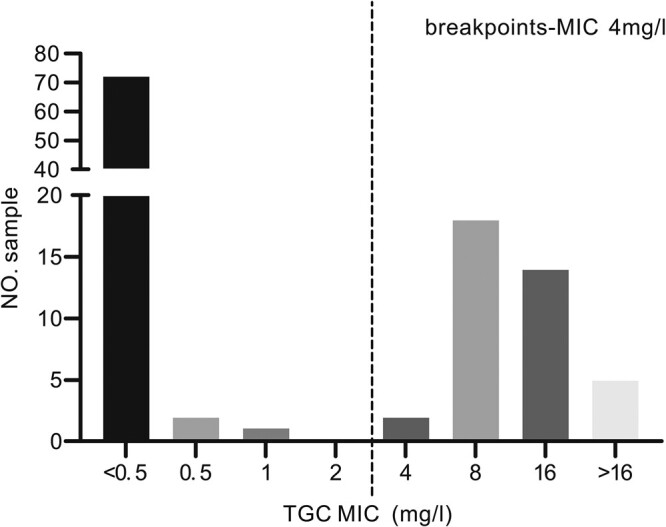
The distribution map of the MIC of tigecycline (TGC) of *E. coli* isolates. In this study, 116 *E. coli* isolates were selected for the verification of the model, of which 72 had a MIC <0.5 mg/L; 2, 1, 0, 2, 18 and 14 had MICs equal to 0.5 mg/L, 1 mg/L, 2 mg/L, 4 mg/L, 8 mg/L and 16 mg/L, respectively; and 5 had MICs >16 mg/L. The TGC breakpoint corresponds to a MIC of 4 mg/L.

For validation, 116 of *E. coli* isolates were selected with MIC values of tigecycline ranging from 0.25 mg/L to 16 mg/L. *tet*(X4) was detected in 96 isolates, whereas no *tet*(X4) was detected in the rest 20 isolates. Fifty-seven isolates of the total 96 were *E. coli*, all of which carried the *tet*(X4) gene. However, their MIC values were all ≤0.5 mg/L. Of the 97 *tet*(X4)-positive *E. coli* isolates, 37 were tigecycline resistant, 2 were intermediate, and 57 were susceptible (Figure 2).

### Analysis of isolates

**Figure 3.**
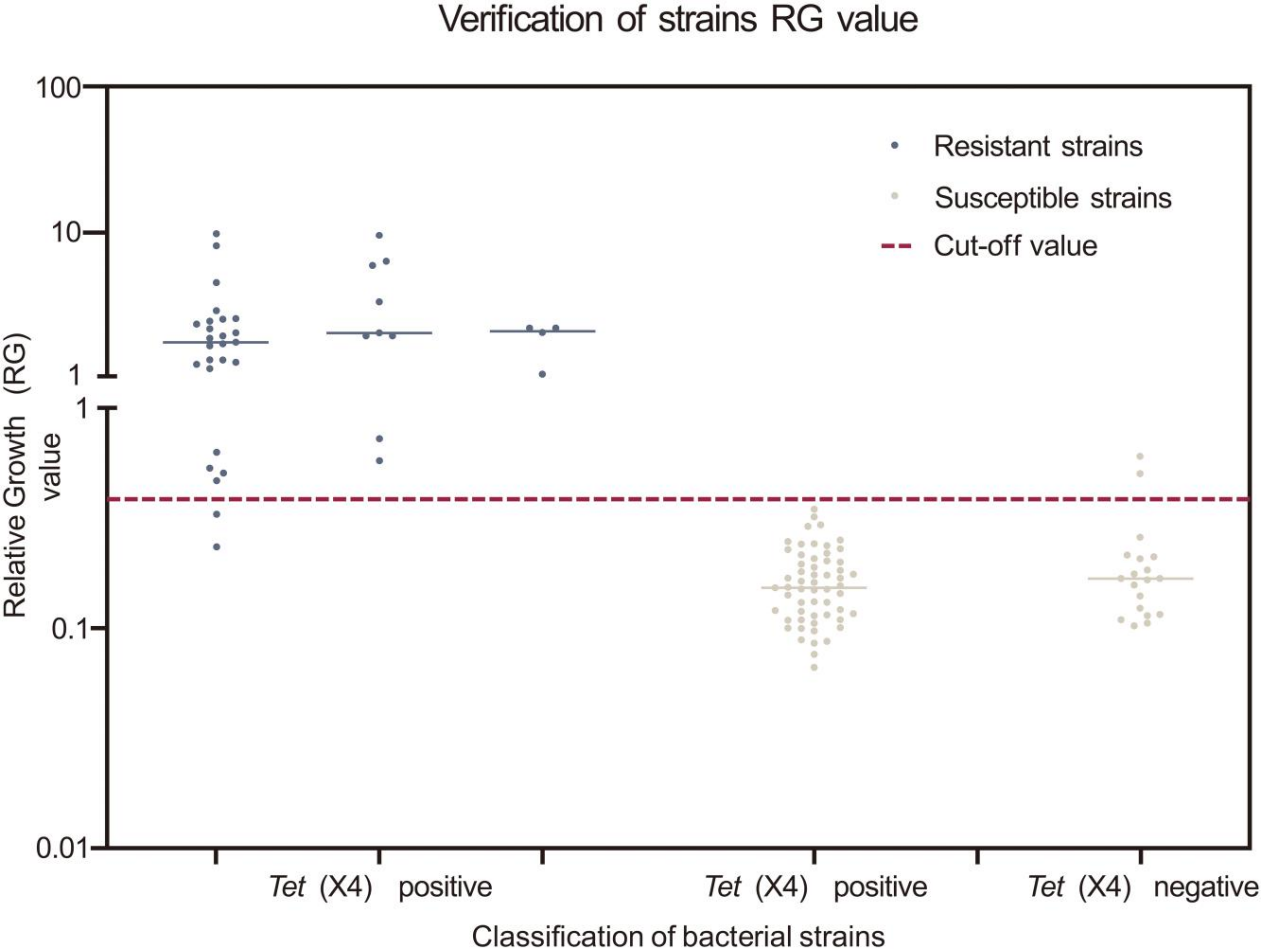
Map of distribution of relative growth (RG) values of verification *E. coli* isolates. In the validation test of 116 *E. coli* isolates. The cut-off value is the RG value of *E. coli* when the susceptible and resistant isolates reach the optimal separation rate.

For confirmation of these results, 116 isolates comprising 96 *tet*(X4) producers and 20 non-producers were analysed. Mass spectrometric results obtained from incubating these isolates with 4 mg/L tigecycline for 2 h confirmed the reliability of the cut-off value obtained semi-quantitatively. The 116 isolates of *E. coli* underwent verification via PCR, comprising the 96 isolates that produced *tet*(X4) and the 20 isolates that did not. Of the 96 *tet*(X4)-positive isolates, 39 were resistant to TGC whereas 57 were not. The drug-resistant phenotypes of 3 of the 96 isolates were incorrectly interpreted. Seventeen isolates of the 20 non-*tet*(X4) producers met the MIC values, whereas the remaining 3 did not. The RG values of 116 *E. coli* isolates in Figure 3. Subsequently, the five isolates whose test results did not match the resistance phenotype were tested twice in duplicate, under the same conditions. The results of both experiments were consistent with the resistance phenotype. This excluded errors caused by the experimental design itself. Disregarding the repeated tests, a susceptibility of 94.9% (95% CI: 81.4%–99.1%) and a specificity of 96.1% (95% CI: 88.3%–99.0%) were obtained using this approach. The results obtained by MALDI-TOF MS combined with the AUC and broth dilution method were statistically analysed, with a kappa value of 0.904. The value shows that the MALDI method agrees with the gold standard method (in general, a kappa value >0.75 is considered excellent agreement; 0.4–0.75 is considered good agreement, and <0.4 is considered poor agreement).^27^

## Discussion

We have demonstrated several key features of using MALDI-TOF MS for pathogen identification and AST analysis. First is the prominence of MALDI in recent commercial assays and its potential for further acceleration. With the continuous development of DNA sequencing technology and the continued reduction in sequencing costs, next-generation sequencing has been widely used in the study of bacterial genomes.^22^ The same is true for MALDI-TOF MS. Whereas the sample preparation steps limited the utility and workflow of previous generations of MALDI, the combined MALDI-TOF MS and AUC approach suggests that these gaps may become smaller in the near future. For example, shorter bacterial culture times, improved experimental steps, and digitization of bacterial growth may help bridge these gaps. As the workflow shifts from traditional culture-based microbiology to molecular detection-based microbiology, MALDI's increased compatibility with phenotypic assays may become an important innovation. Specifically, this enhanced integration of MALDI with phenotypic testing could be a vital development.

This research shows a new approach to tigecycline resistance detection in *E. coli*, the progress in reducing the time to results, and the quality of the results. It amply verifies the feasibility of using the statistical method of MS combined with the AUC to analyse the resistance phenotype of *tet*(X4) producers and non-*tet*(X4) producers, which significantly improves on previous analysis of *tet*(X4)-resistant isolates only. Previous reports have focused on tigecycline resistance in animals and their environment.^2,28–30^ However, understanding and coping with the spread of tigecycline resistance should be cited, and our assay should also be generalizable to human isolates.

One of the major advantages of our study is a significant reduction in laboratory reporting time for identifying tigecycline resistance phenotypes in *E. coli*. From preliminary preparation, bacterial incubation, bacterial profiling and result analysis, the process can be completed in 3 h. This then leads to a second advantage. Clinical experiments can be conducted with little reagent consumption if the operation is simple and quick, as well as reducing some medical expenses by eliminating the need for test strips and culture plates that are required in AST. Our study paves the way for the detection of resistance phenotypes of bacteria of the same or different species to the same or different drugs, a topic that needs further research.

In the current study, we unveiled the limitations of phenotypic detection and revealed various aspects of susceptibility testing by exploiting MALDI-TOF MS and AUC assays. Generally, genes are linked to phenotypes and diseases through epigenetics. In spite of this, it remains difficult to determine precisely what the relationship between genotype, residual enzyme activity and clinical epigenetics is.^31^ The same is also true of studies looking at resistance to tigecycline in *E. coli*. For the tigecycline-susceptible phenotype exhibited by *tet*(X4)-positive *E. coli*, we speculate whether an azidothymidine-like DNA damage mechanism or a potential *tet*(X4) inhibitor exists within *E. coli.*^32^ The applicability of the experiment has been demonstrated for a variety of different bacterium-antibiotic combinations.^23^ Coupled with this, we have developed a method to determine bacterial growth under antibiotic inhibition. Specifically, MS technology is used to demonstrate the characteristic peaks of bacteria. The number of these peaks is then quantified via R software and AUC analysis. This approach is well suited for large-scale clinical screening of antimicrobial resistance phenotypes in targeted bacterial species. Moreover, it is rapid, convenient and readily implemented in clinical laboratories already possessing MALDI-TOF MS equipment.

### Conclusion

In summary, this approach shows how methods based on MALDI-TOF MS provide an opportunity for implementation in other Gram-negative bacteria. Our approach brings a new insight, namely applying MALDI-TOF MS with the AUC method to detect tigecycline resistance among *E. coli* isolates. This novel approach has several significant advantages over conventional susceptibility testing methods in terms of convenience and accuracy. Besides, our results highlight the importance of monitoring the spread of antimicrobial resistance in the environment, especially in the context of the One Health perspective. As a bacterial species that exists widely in humans, animals and the environment, the high prevalence of *tet*(X4)-positive *E. coli* raises the risk of antimicrobial resistance transmission. In addition, the method is suitable for large-scale clinical screening of antibiotic resistance phenotypes of certain bacteria: it is convenient, quick and easy to promote for clinical labs already equipped with MALDI-TOF MS.
